# The evolutionary logic of socialized older population care service demand in rural areas based on the HAPC model

**DOI:** 10.3389/fpubh.2026.1745568

**Published:** 2026-01-27

**Authors:** Hongyu Chen, Lilong Zhang, Xiaojing Han, Xueyuan Chen, Shanwei Li, Yongchang Wu

**Affiliations:** 1 Institute of Agricultural Economics and Development, Chinese Academy of Agricultural Sciences, Beijing, China; 2 School of Labor Economics, Capital University of Economics and Business, Beijing, China

**Keywords:** CLHLS data, hierarchical age–period–cohort (HAPC) model, rural older population demand, socialized older population care services, trend analysis

## Abstract

Chinese rural areas have entered a stage of moderate population aging ahead of urban regions, where mismatches between the supply and demand of older population care services are particularly pronounced. Establishing a socialized older population care service system has therefore become a critical response to the challenges posed by rural aging. This study develops a theoretical framework to explain the demand generation mechanism for rural socialized older population care services, identifying key influencing factors under both policy-driven and individual choice scenarios. Using data from the China Longitudinal Healthy Longevity Survey (CLHLS) from 2005 to 2018, we employ a hierarchical age–period–cohort (HAPC) model to examine the effects of age, period, and cohort on service demand. The results show that period effects are characterized by fluctuating upward trends in demand for both basic and expanded care services, primarily driven by changes in policy environments and economic conditions. Cohort effects significantly shape demand for expanded care services, but not for basic care services, with notable heterogeneity across regions and gender groups. Overall, rural demand for socialized older population care services is jointly influenced by age, period, and cohort dynamics. These findings suggest that optimizing the supply of older population care services in rural areas requires better alignment with differentiated demand characteristics to improve service relevance and effectiveness.

## 1 Introduction

Over the past decade, population aging in China’s rural areas has proceeded at a markedly faster pace than in urban regions. According to the Seventh National Population Census (2020), the rural population aged 60 and above reached approximately 121 million, accounting for 23.81% of the total rural population, compared with only 14.98% in 2010. Moreover, the United Nations World Social Report 2023 projects that by 2050, China’s population aged 65 and above will reach 390 million, representing 24.64% of the global older population population, making China the country with the largest older population population worldwide. According to internationally recognized aging standards issued,^1^ China’s rural areas have already entered the stage of moderate aging, facing significantly greater older population care challenges than urban areas. However, the existing socialized older population care service system in rural China remains insufficient to meet the rapidly growing and increasingly diversified needs of the aging population.

The sustained outflow of young and middle-aged rural residents, together with deeply rooted traditional care services for the older population norms, has further intensified pressures on rural older population care systems. The 2024 National Migrant Worker Monitoring Survey Report shows that the total number of migrant workers nationwide reached 299.73 million, with 178.71 million working outside their home regions—an increase of 1.2% from the previous year. Meanwhile, the China Population Mobility Forecast Report projects that by 2040 China’s permanent resident population will approach 1.345 billion, with an urbanization rate of 78.6%. Most of the newly urbanized population is expected to concentrate in metropolitan areas and urban agglomerations, suggesting that the outflow of rural labor will persist over the long term. As a result, the phenomenon of left-behind older population individuals in rural areas is likely to become increasingly pronounced.

The sustained outflow of young and middle-aged rural residents, together with deeply rooted traditional care services for the older population norms, has further intensified pressures on rural older population care systems. The 2024 National Migrant Worker Monitoring Survey Report shows that the total number of migrant workers nationwide reached 299.73 million, with 178.71 million working outside their home regions—an increase of 1.2% from the previous year. Meanwhile, the China Population Mobility Forecast Report projects that by 2040 China’s permanent resident population will approach 1.345 billion, with an urbanization rate of 78.6%. Most of the newly urbanized population is expected to concentrate in metropolitan areas and urban agglomerations, suggesting that the outflow of rural labor will persist over the long term. As a result, the phenomenon of left-behind older population individuals in rural areas is likely to become increasingly pronounced.

In addition, the traditional belief that “children are the insurance for old age” remains deeply entrenched among rural older population residents (1, 2). Many older adults continue to view their children as the primary providers of care services for the older population, demonstrating resistance to institutionalized care arrangements and a strong preference for home-based care. At the same time, transformations in family structure have made the “4-2-2” model—where a couple supports four older population parents while raising two children—increasingly common. Under mounting work and family pressures, younger generations often struggle to provide adequate in-home care for older population relatives, leading to a continued decline in traditional family caregiving functions (3). Against this backdrop, rural socialized older population care systems exhibit persistent structural deficiencies, including mismatches between supply and demand, weak resource mechanisms, and fragmented service provision, limiting their capacity to respond effectively to rapidly expanding care needs.

As a result, the combined effects of rural labor outmigration, weakening family caregiving functions, and underdeveloped socialized older population care systems have pushed rural older population care to the forefront of public and policy concern. In February 2022, the State Council issued the 14th Five-Year Plan for the Development of the Older population Care Service System, explicitly identifying imbalance and inadequacy as central problems in China’s older population care services, with underdeveloped rural provision representing a key bottleneck. Subsequently, the establishment of a comprehensive rural older population care service network was incorporated into the Opinions of the Central Committee of the Communist Party of China and the State Council on Deepening the Reform and Development of Older population Care Services. More recently, the 2024 Guiding Opinions on Accelerating the Development of Rural Older population Care Services proposed concrete measures, including the construction of township-level regional older population care service centers and the expansion of village-level service points.

From a theoretical perspective, the concept of “socialized older population care services” was formally discussed as early as 1998 at the National Symposium on Family-Based Elder Care and Socialized Elder Care Services, which examined its definition, prerequisites, and developmental trajectory. As China’s demographic structure and older population care needs have continued to evolve, scholars have progressively clarified its core connotation: socialized older population care seeks to ensure older population individuals’ basic living rights—including economic security, daily care, and emotional support—through socially organized systems that mobilize diverse resources (4). Its fundamental logic lies in transferring the responsibility for care services for the older population from families to society, with services delivered through purchased or publicly provided mechanisms (5, 6).

Building on this foundation, two dominant perspectives have emerged in the literature. The first emphasizes multi-actor collaboration, arguing that socialized older population care services are jointly provided by governments, communities, social organizations, and market actors, with family-based care, community-based care, and institutional care integrated into a comprehensive service model (7). The second highlights the necessity of socialized older population care as a transformative departure from traditional family-based care, representing a diversification of care services for the older population pathways beyond a single dominant model (8). In the context of rapid urbanization, the simultaneous emergence of rural “hollowing-out” and urban “empty-nesting” phenomena has placed disproportionate pressure on rural older population care systems (9), leading to development trajectories that differ substantially from those observed in urban areas. Nevertheless, rural communities often possess dense social networks and strong relational familiarity, which facilitate the formation of community governance structures and provide favorable conditions for mutual-aid and community-based care services for the older population models (10). Community-based home care can therefore improve both the material and emotional well-being of rural older population residents while alleviating caregiving burdens on younger generations.

Despite growing policy attention and academic interest, existing studies remain limited in explaining why and how demand for socialized older population care services is formed in rural China. First, much of the literature relies on single-dimensional, static analyses, with insufficient attention to the dynamic evolution of individual demand across different life-course stages. Second, research examining the complex interaction between policy-driven factors and individual choice in rural contexts—and quantitatively capturing this dual influence using the age–period–cohort framework—remains scarce.

Moreover, existing studies often adopt overly broad definitions of socialized older population care services, lacking clear service boundaries and hierarchical distinctions. To address these limitations, this study defines rural socialized older population care services as a composite, under government leadership and with active participation from non-governmental organizations, this integrated service system aims to deliver essential services (such as daily living assistance and medical care) and supplementary services (such as emotional support and cultural activities) to all older population residents in rural communities through either purchase or free provision. This definition not only distinguishes socialized older population care from traditional family-based and mutual-aid models but also establishes a solid theoretical foundation for subsequent empirical analysis.

Accordingly, this study seeks to address two core research questions. First, how can a theoretical framework be constructed to explain the internal mechanisms through which demand for rural socialized older population care services is generated, integrating individual life-course experiences with macro-level policy evolution? Second, under the combined influence of policy drivers and individual choices, how can the age–period–cohort framework be employed to isolate the net effects shaping rural older population demand for socialized care services, thereby informing policy design and supply optimization?

Given the inherent identification problem in traditional age–period–cohort models caused by perfect multicollinearity among age, period, and cohort (11), this study adopts a hierarchical age–period–cohort (HAPC) model, which belongs to the class of hierarchical random cross-effects models (12). By treating individual age as a first-level variable and survey period and birth cohort as second-level random effects, the HAPC approach effectively mitigates multicollinearity concerns. Specifically, the model allows for the separate identification of: (1) age effects, capturing physiological and behavioral changes in care demand as individuals age; (2) period effects, reflecting the common influence of macro-level policies, economic conditions, and external shocks during specific survey years; and (3) cohort effects, representing long-term preferences and value orientations shaped by shared historical experiences. This hierarchical modeling strategy enhances estimation robustness and provides a rigorous quantitative framework for examining the interaction between institutional supply and individual agency in shaping demand for older population care services. In doing so, it offers both theoretical insights and policy guidance for advancing rural modernization and building a high-quality rural older population care service system.

## 2 Theoretical analysis and research hypotheses

Life course theory offers a multidimensional and systematic analytical framework for understanding the dynamic evolution of rural older population individuals’ demand for socialized older population care services. The theory emphasizes that individual life trajectories do not unfold in isolation but are embedded within specific historical contexts and social structures (13). From this perspective, conditions in old age and corresponding service needs are shaped not only by physiological aging, but also by early-life experiences, exposure to particular historical periods, and generational positioning. Accordingly, variations in rural seniors’ demand for socialized older population care services can be understood through three core dimensions: age effects, period effects, and cohort effects, which, respectively, capture the influences of individual life-cycle progression, macro-level policy and socioeconomic changes, and cohort-specific characteristics.

Within this framework, the mechanism of cumulative disadvantage constitutes a central proposition of life course theory. Individuals who experience structural disadvantages early in life—such as poverty, limited access to education, inadequate health investment, or exposure to social instability—tend to accumulate health-related vulnerabilities and deficits in social capital. These disadvantages are not temporary but compound over time, ultimately leading to substantial erosion of human and health capital in later life. Such cumulative processes significantly increase reliance on high-quality socialized older population care services in old age (14). This mechanism is particularly salient for explaining the widespread vulnerability in older population care observed among rural populations in China, where long-standing socioeconomic disparities continue to shape aging outcomes.

### 2.1 Age effects and phase-specific differentiation in older population care service demand

The age effect suggests that individual behavior evolves systematically with advancing age as life trajectories, life stages, and social roles change. At the same time, individuals’ choices and interactions within their social environments continuously shape their life-course pathways (15). In the context of rural older population populations, increasing age is often accompanied by declining physical capacity and heightened dependence on family support, reinforced by deeply rooted norms of family-based care services for the older population. As a result, older seniors tend to favor home-based care arrangements that provide basic and personalized older population care services. By contrast, younger seniors generally retain stronger self-care abilities and exhibit greater openness to emerging care models. Coupled with more diversified aspirations for quality of life, they are more likely to demand expanded older population care services beyond basic daily assistance.

*H1*: Demand for basic older population care services increases with age, whereas demand for expanded older population care services decreases with age.

### 2.2 Period effects and policy-driven structural shaping of older population care service demand

Period effects capture the collective influence of macroeconomic conditions, institutional arrangements, and public policy changes at specific historical junctures on the entire population. In recent years, the Chinese government has continuously advanced the development of rural older population care service systems through a sequence of policy interventions, gradually establishing an institutionalized supply framework that has substantially reshaped both the accessibility of services and rural residents’ cognitive recognition of socialized older population care.

A clear policy shift emerged in 2011, when the State Council issued the Social Older population Care Service System Development Plan, which for the first time introduced the concept of “new rural mutual-aid older population care.” Subsequently, in 2015, the Central Office of the Communist Party of China and the State Council jointly launched pilot programs for rural community-based older population care, explicitly promoting a service model that integrates mutual-aid and community-based care. This model emphasized multi-source resource integration, characterized by government guidance, support from village collectives, and participation of social organizations. In 2019, the CPC Central Committee and the State Council released the National Medium- and Long-Term Plan for Actively Responding to Population Aging, formally incorporating rural older population care into the national older population care service system. The plan proposed the construction of a comprehensive system featuring complete functions, appropriate scale, urban–rural integration, and coordinated development of medical and older population care services.

Most recently, in 2024, the Ministry of Civil Affairs, together with 21 other departments, issued the Guiding Opinions on Accelerating the Development of Rural Older population Care Services. As China’s first comprehensive national-level policy document specifically targeting rural older population care, its promulgation marked a new stage in the development of rural socialized older population care services—signaling a transition from fragmented, exploratory initiatives to systematic and coordinated institutional integration. The policy’s intensity of capacity building and depth of institutional design are unprecedented in the history of rural older population care development in China.

Taken together, this evolving policy framework has not only progressively addressed long-standing gaps in rural older population care service provision, but also functioned as a critical institutional safeguard against the continued erosion of family-based caregiving capacity in rural areas. By responding to the structural pressures of population aging, these policies provide a viable and increasingly standardized pathway for rural older population residents to access diversified and higher-quality care services.

*H2*: As the rural older population care policy framework matures over time, overall demand for socialized older population care services among rural seniors increases. Moreover, policy effects are stronger for demand for supplementary services than for basic services, reflecting shifts in care perceptions and service expectations.

### 2.3 Cohort effects and intergenerational differences in older population care service demand

The cohort effect highlights that individuals belonging to the same birth cohort tend to develop relatively stable behavioral patterns and value orientations over the life course as a result of shared socio-historical experiences (13, 15). Rural older population individuals from different generations were exposed to markedly distinct socioeconomic environments during their formative years. These early-life conditions shaped their resource endowments, family structures, and levels of trust in public institutions, which in turn influenced their attitudes toward and preferences for socialized older population care services in later life.

For example, rural elders born before 1900 came of age in the late Qing dynasty and the early Republican period, a time marked by war, political instability, and foreign aggression. During this era, rural public services and formal social security systems were virtually absent, leaving survival in old age largely dependent on traditional family support and clan-based mutual aid networks. While such experiences fostered strong self-reliance and resilience, they were also associated with severe constraints on overall living conditions and long-term human capital accumulation.

By contrast, rural seniors born after 1946 grew up following the founding of the People’s Republic of China and generally benefited from land reform and the establishment of collective institutions during their youth. Although this cohort typically attained relatively low levels of formal education, its members tend to exhibit strong collectivist values and a pronounced sense of national identity (16). As socialized older population care services gradually emerged in rural areas—alongside increasing pressures on family-based caregiving—this generation became more receptive to institutional and community-based care arrangements. Consequently, they have shown a growing willingness to engage with socialized older population care services and to adapt to a hybrid caregiving model that combines family support with broader societal provision.

*H3*: Compared with earlier birth cohorts, later-born rural older population cohorts exhibit significantly higher demand for expanded older population care services, reflecting their socialization in environments with progressively expanding public service provision and smaller family structures. In contrast, no significant cohort differences are expected in demand for basic older population care services.

This study examines heterogeneity in older population care service demand primarily through the lenses of gender theory and theories of uneven regional development. Gender theory emphasizes that gender roles are socially constructed and embedded within long-standing divisions of labor. Men, traditionally positioned as primary economic providers within the family, often experience a compression of personal care needs due to sustained family and work responsibilities. Women, historically socialized as family caregivers, tend to prioritize household and caregiving duties over their own needs. As a result, gender-differentiated care demands often remain latent and only gradually surface in later life, particularly during structural transitions such as the emergence of “empty-nest” households. This process constitutes the core theoretical logic underlying gender-based heterogeneity in older population care service demand.

The theory of uneven regional development further explains spatial heterogeneity in older population care service demand by highlighting disparities in economic foundations and public service provision across regions. Rural areas in eastern China, benefiting from greater resource concentration and stronger fiscal capacity, initiated the provision of diversified older population care services at an earlier stage, leading to earlier and more active demand formation and adjustment. By contrast, rural areas in western and northeastern China, constrained by weaker economic bases and limited public service resources, have historically experienced suppressed demand for socialized older population care services. It is only with the gradual infusion of policy-driven resources that such latent demand has begun to be released. Together, these mechanisms provide a robust theoretical foundation for understanding regional heterogeneity in rural older population care service demand.

## 3 Variable configuration and model setup

### 3.1 Data sources

The data used in this study are drawn from the China Longitudinal Healthy Longevity Survey (CLHLS), which is jointly organized by Peking University and relevant research institutions. The survey covers 23 provinces, municipalities, and autonomous regions across China and targets individuals aged 65 and above. Data are collected through structured questionnaires administered both to surviving older population respondents and, in cases of death, to close family members acting as proxy respondents. As an open-access public database, the CLHLS provides nearly two decades of continuous, nationally representative micro-level data and has been widely used in aging-related research.

The CLHLS is particularly well suited to this study due to its inclusion of key variables relevant to older population care service demand, its extended temporal coverage, and the consistency of questionnaire design across survey waves. Considering the relatively poor data quality of the 1998 wave and the absence of relevant variables in surveys conducted prior to 2002, this study utilizes data from five survey waves conducted between 2005 and 2018. After sample selection and data cleaning, a total of 30,585 valid observations were retained for empirical analysis.

### 3.2 Variable settings

#### 3.2.1 Dependent variable

The dependent variable in this study is rural older population individuals’ demand for socialized older population care services. This demand is defined as the subjective need for care services provided by governments, communities, and other social actors within a community-based framework, reflecting older adults’ health status, self-care capacity, and willingness to engage in social participation. The data are derived from Item F15 of the China Longitudinal Healthy Longevity Survey (CLHLS) questionnaire, which covers six categories of older population care services: (1) daily living assistance; (2) home medical visits and medication delivery; (3) emotional support and companionship; (4) daily shopping assistance; (5) organized social and recreational activities; and (6) health education provision. For each service item, respondents reported whether they required the service (“yes” or “no”). After excluding observations with missing values, valid responses for all six service categories were retained for analysis.

Following Hu (17) definition of basic older population care services as comprehensive services covering fundamental daily care, long-term care for disability and dementia, emotional support, and emergency assistance, this study classifies the six service items into two categories. Specifically, daily living assistance, home medical visits and medication delivery, and emotional support and companionship are defined as basic older population care services, while daily shopping assistance, organized social and recreational activities, and health education provision are categorized as expanded older population care services. Operationally, if a respondent reported demand for at least one service item within a given category, the corresponding category variable was coded as 1; otherwise, it was coded as 0.

#### 3.2.2 Core independent variables

The core independent variables are age, period, and birth cohort. The analytical sample includes individuals aged 65–119 years, observed in five survey waves conducted in 2005, 2008, 2011, 2014, and 2018. Birth cohorts span from 1885 to 1953. In the hierarchical age–period–cohort (HAPC) model, age is treated as a continuous individual-level variable. To enhance parameter interpretability, reduce multicollinearity, and improve estimation efficiency and stability, age is mean-centered prior to analysis. Period is defined by the CLHLS survey year and is modeled as a random effect. Birth cohort is conceptually continuous; however, based on the distribution of birth years, historical context, and computational considerations, cohorts are grouped into seven categories corresponding to major historical periods: ≤1900, 1901–1911, 1912–1918, 1919–1927, 1928–1936, 1937–1945, and ≥1946.

#### 3.2.3 Control variables

The control variables include Activities of Daily Living (ADL) ability (continuous variable, with higher values indicating poorer ADL functioning); geographic region (Eastern = 1, Central = 2, Western = 3, Northeast = 4); gender (male = 1, female = 0); educational attainment (schooled = 1, unschooled = 0); marital status (married = 1, other = 0); primary source of income (self-generated income = 1, other sources = 0); living arrangement (living with family = 1, living alone = 0); quality of life (categorical variable, with higher values indicating lower quality of life); household economic status (categorical variable, with higher values indicating poorer economic conditions); and number of children ever born (continuous variable).

### 3.3 Model configuration

This study constructs a hierarchical age–period–cohort (HAPC) model with demand for socialized older population care services as the dependent variable, in order to disentangle the effects of age, period, and cohort on rural older population individuals’ service demand while controlling for temporal influences across multiple dimensions. By incorporating age at the individual level and period and cohort at higher levels, the model enables a systematic examination of how life-course dynamics and macro-level temporal factors jointly shape demand patterns.

Model estimation is conducted using SAS 9.4, and graphical analyses of results are performed in Microsoft Excel. Given that the dependent variable is binary, the analysis adopts a generalized linear hierarchical mixed-effects modeling framework with an appropriate link function. Taking demand for basic socialized older population care services as an illustrative case, the formal specification of the HAPC model is presented as follows.

First-level model (individual level):

$${\mathrm{BD}}_{\text{Yijk}=1}=\beta_{0\mathrm{jk}}+\beta_{1}\mathrm{\alpha g}e_{\mathrm{ijk}}+\sum_{p=7}^{p}\beta_{p}{\mathrm{CV}}_{\mathrm{ijk}}$$ (1)

Second-level model (group level):

$$\beta_{0\mathrm{jk}}=\gamma_{0}+\mu_{0j}+\nu_{0k},\mu_{0j}∼N(0,\tau_{\mu 0}),\nu_{0k}∼N(0,\tau_{g0})$$ (2)

Combined model:

$${\mathrm{BD}}_{\text{Yijk}=1}=\beta_{1}\mathrm{\alpha g}e_{\mathrm{ijk}}+\sum_{p=7}^{p}\beta_{p}{\mathrm{CV}}_{\mathrm{ijk}}+\gamma_{0}+\mu_{0j}+\nu_{0k}$$ (3)

In Equation 1, ${\mathrm{BD}}_{\text{Yijk}=1}$ denotes individual i’s high demand for basic older population care services during period j (survey year) and generation k. ${\mathrm{age}}_{\mathrm{ijk}}$ represents the individual’s age; ${\mathrm{CV}}_{\mathrm{ijk}}$ represents control variables, which in this study comprise seven covariates influencing demand for older population care services, including gender, educational attainment, and living conditions. $\beta_{0\mathrm{jk}}$ is the intercept, representing the average level of demand for basic older population care services among individuals of generation k in survey year j. $\beta_{1}$ - $\beta_{p}$ is the primary fixed effect.

In Equation 2, $\gamma_{0}$ is the model intercept, representing the expected mean when all first-level variables are zero across all periods and cohorts; $\mu_{0j}$ is the period j average random effect across all birth cohorts, following a normal distribution with variance $\tau_{\mu 0}$ ; $\nu_{0k}$ is the cohort k average random effect across all survey periods, also following a normal distribution with variance $\tau_{g0}$ .

## 4 Empirical results analysis

### 4.1 Analysis of basic older population care service demand

The estimation results of the hierarchical age–period–cohort (HAPC) model for demand for basic older population care services are reported in Table 1. The table is divided into two sections. The first section presents the fixed-effects estimates, which capture the effects of individual-level characteristics on rural older population individuals’ demand for basic older population care services. The second section reports the variance components of the random effects, which assess the extent to which period and cohort factors contribute to variations in demand at the higher level.

**Table 1:** Estimation results of the HAPC model for basic older population care service demand.

Variable Name	Coefficient	Standard deviation	*p*-value
Fixed effects			
Intercept	0.2260	0.1908	0.3018
Age	0.0059	0.0019	0.0019
Self-care ability	0.0078	0.0028	0.0050
Region (reference group: northeast)			
Eastern	0.8662	0.0642	<0.0001
Central	0.6694	0.0669	<0.0001
Western	0.7174	0.0654	<0.0001
Gender (reference group: male)	0.0080	0.0359	0.8237
Educational attainment (reference group: schooled)	0.0294	0.0370	0.4266
Marital status (reference group: married)	0.3218	0.0458	<0.0001
Primary source of income (reference group: self-generated income)	−0.1037	0.0402	0.0098
Living arrangements (reference group: family)	0.1714	0.0400	<0.0001
Quality of life	0.0981	0.0209	<0.0001
Household financial status	0.0670	0.0256	0.0088
Number of children born	−0.0078	0.0071	0.2741
Random effects variance			
Period	0.1170	0.0837	0.0811
Generation	0	–	–
−2LogLikelihood	146932.0		

Positive coefficient estimates indicate that the corresponding explanatory variable increases the odds of reporting demand for basic older population care services, whereas negative coefficients imply a lower likelihood. At the individual level, age, Activities of Daily Living (ADL) ability, region, marital status, primary source of income, living arrangement, quality of life, and household economic status are all statistically significant at the 5% level or above, indicating that these factors exert significant influences on demand for basic older population care services. In contrast, gender, educational attainment, and number of children do not reach statistical significance at the 5% level, suggesting that these variables have no significant independent effect on demand in this model.

At the second level, the variance components for both period and cohort effects fail to pass the 5% significance test. This indicates that, after controlling for individual-level characteristics, neither period-specific nor cohort-specific random effects exert a statistically significant influence on rural older population individuals’ demand for basic older population care services. In other words, demand for basic services appears to be primarily driven by individual-level factors rather than by temporal or generational heterogeneity.

#### 4.1.1 Age effect

Age exerts a positive linear effect on rural seniors’ demand for basic older population care services. As age increases, the likelihood of demanding basic care services exhibits a sustained upward trend. In the fixed-effects estimation of the HAPC model for basic older population care service demand (Table 1), the coefficient on age is 0.0059 and is statistically significant at the 1% level (*p* = 0.0019), indicating a robust positive association between age and demand for basic older population care services among rural seniors. Specifically, holding other factors constant, each one-year increase in age is associated with an increase in the probability that an individual reports demand for basic older population care services.

Although the estimated coefficient is relatively small in magnitude, age is a continuous variable with cumulative effects. Consequently, the influence of aging on care demand becomes increasingly salient over the life course, particularly at more advanced ages. This finding is consistent with practical expectations: as individuals grow older, physical functioning and self-care capacity gradually decline, leading to heightened reliance on basic older population care services (18).

To further assess age-related heterogeneity in demand for basic older population care services, this study introduces interaction terms between age and region, as well as between age and gender. The estimation results are reported in Table 2. With respect to regional heterogeneity, the interaction terms between age and the Central and Western regions are statistically significant at the 5% level, while the interaction between age and the Eastern region is marginally significant at the 10% level. In contrast, the interaction between age and the Northeast region does not reach statistical significance. These findings indicate that regional context partially moderates the age effect on demand for basic older population care services, suggesting the presence of relatively stable but differentiated regional patterns.

**Table 2:** Heterogeneity of age effects on basic older population care service demand among rural seniors.

Variable name	Regional heterogeneity			Gender heterogeneity		
	Coefficient	Standard deviation	*p*-value	Coefficient	Standard deviation	*p*-value
Age × region (eastern region)	0.0045	0.0026	0.0849			
Age × region (central region)	0.0073	0.0031	0.0166			
Age × region (western region)	0.0087	0.0027	0.0016			
Age × region (northeastern region)	−0.0055	0.0053	0.3023			
Age × gender (female)				0.0055	0.0024	0.0220
Age × gender (male)				0.0066	0.0027	0.0154
Control variables	Under control			Under control		

Gender also exhibits a strong moderating effect on the relationship between age and demand for basic older population care services. The interaction between age and gender is highly statistically significant, indicating that the positive effect of age on demand is amplified across both male and female older population populations. In other words, regardless of gender, increases in age strengthen the likelihood of reporting demand for basic older population care services, underscoring the robustness of the age effect while highlighting meaningful gender-based heterogeneity in its magnitude.

#### 4.1.2 Period effect

**Figure 1:**
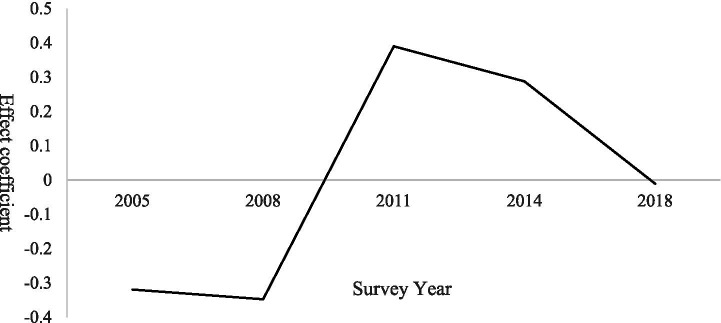
Period effects on demand for basic older population care services among rural seniors.

The period-effect model indicates that demand for basic older population care services follows a pattern of initial increase followed by subsequent decline. As illustrated in Figure 1, demand remains within the negative-effect range and exhibits a slight downward trend from 2005 to 2008. During this phase, accelerated urbanization triggered large-scale outmigration of rural labor, weakening traditional family-based caregiving capacity. Nevertheless, older population care in rural areas continued to rely predominantly on families, while limited public service provision constrained effective demand realization, leading to the accumulation of latent care needs.

Between 2008 and 2011, demand rose sharply and reached a positive peak. This surge was driven by rising rural incomes associated with a series of pro-farmer policies implemented during this period. Policy initiatives—including the 2009 pilot of the New Rural Pension Scheme and the 2010 Older population Care Service System Development Plan—expanded the scope of public older population care provision. At the same time, growing societal attention to the issue of “empty-nest older population” accelerated the release of previously suppressed demand (19). From 2011 to 2018, the period effect gradually declined toward zero. This decline reflects the initial saturation of basic service coverage following earlier policy expansion, as well as partial labor return driven by county-level economic development and the tendency of some older population individuals to relocate and live with their adult children, thereby reducing care demand intensity (20).

**Figure 2:**
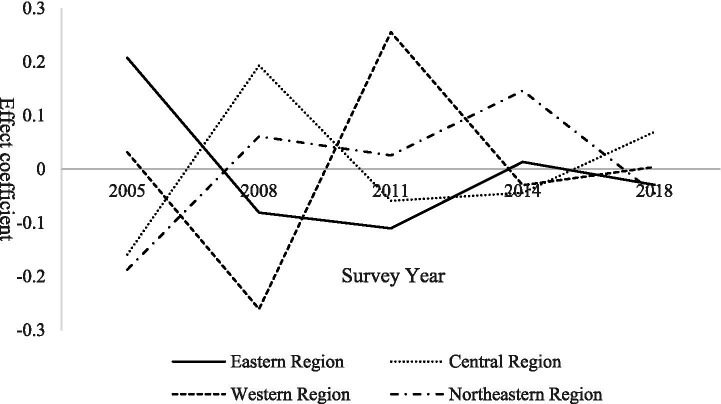
Regional heterogeneity in period effects on demand for basic older population care services among rural seniors.

Further analysis of period effects and group heterogeneity reveals that regional differences play a significant explanatory role, with distinct temporal demand trajectories observed across regions (21). In contrast, gender heterogeneity does not yield statistically significant differences in period effects, indicating that the temporal evolution of demand for basic older population care services is broadly similar for male and female seniors (22). The results of regional heterogeneity analysis are presented in Figure 2.

In the eastern region, the period effect declined rapidly between 2005 and 2008 before gradually rebounding. This pattern reflects early urbanization and substantial labor outmigration, which weakened family-based care in the initial stage. Subsequently, supported by stronger economic foundations, the region experienced partial labor return and prioritized the development of supporting older population care services, thereby easing demand pressure. The central region exhibited a sharp increase in demand during 2005–2008, followed by fluctuating declines. As a major source of migrant labor, the pronounced “empty-nest” phenomenon initially released substantial demand, which was later mitigated as policy interventions expanded basic service coverage. In the western region, demand rebounded gradually after reaching a low point during 2005–2008. This rebound reflects the release of long-suppressed demand caused by earlier economic constraints and insufficient care supply, which was partially alleviated by poverty alleviation programs that strengthened older population care infrastructure. In contrast, the northeastern region shows a steady upward trend in demand over time, driven by sustained population outflow and continued erosion of family-based caregiving. However, the relatively slow expansion of older population care services amid regional economic restructuring has contributed to increasing demand pressure.

#### 4.1.3 Cohort effect

**Figure 3:**
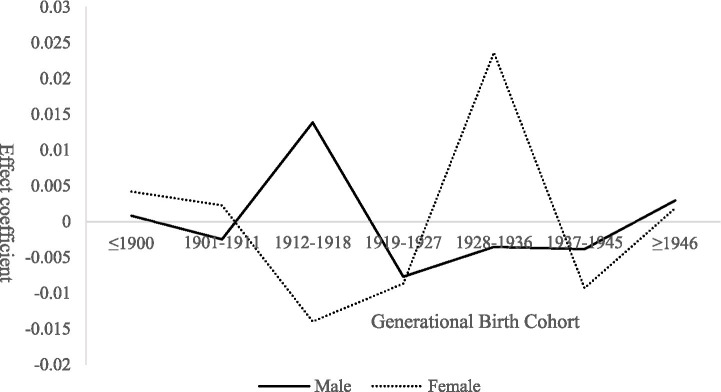
Gender heterogeneity in cohort effects on demand for basic older population care services among rural seniors.

Although the generational effect on demand for basic older population care services is not statistically significant overall, pronounced gender heterogeneity is observed within cohort effects (23), as illustrated in Figure 3. For males, the cohort effect exhibits a pattern of “wave-like adjustment” across birth cohorts: slightly positive among those born ≤1900, approximately zero for the 1901–1911 cohort, an increase followed by a decline for the 1912–1918 cohort, and a gradual upward trend thereafter. This pattern is closely related to cohort-specific family roles and social contexts. Earlier cohorts, who typically dominated household decision-making, tended to internalize care needs, rendering demand less explicit. Middle cohorts experienced substantial transitions in family care functions amid social transformations—such as collectivization and labor mobility—leading to corresponding fluctuations in care demand. For later cohorts, the intensification of aging-related constraints contributed to increasingly explicit demand for basic care services.

In contrast, cohort effects among females display greater volatility. Demand is slightly positive for those born ≤1900, followed by a sharp decline and subsequent recovery for the 1901–1911 cohort, peaking among those born between 1928 and 1936 before declining thereafter. This trajectory reflects gendered caregiving dynamics across generations. Women in earlier cohorts, bearing primary family caregiving responsibilities, often experienced their own care needs being overlooked. Among middle cohorts, increased social participation combined with shrinking family size facilitated a rapid release of latent demand—particularly for daily assistance and emotional support. For later cohorts, the gradual expansion and normalization of basic service coverage reduced marginal demand intensity over time.

### 4.2 Analysis of extended older population care service demand

The estimation results of the hierarchical age–period–cohort (HAPC) model for demand for expanded older population care services are reported in Table 3. At the individual level, self-care ability, region, educational attainment, marital status, living arrangement, quality of life, and number of children born all exhibit statistically significant effects at the 5% level, indicating that these factors play important roles in shaping rural older population individuals’ demand for expanded care services. In contrast, age, gender, primary source of livelihood, and household economic status do not reach statistical significance at the 5% level, suggesting that these variables have no independent effect on demand for expanded older population care services in this model.

**Table 3:** Estimation results of the HAPC model for demand for expanded older population care services.

Variable name	Coefficient	Standard deviation	*p*-value
Fixed effects			
Intercept	0.8634	0.1741	0.0077
Age	−0.0018	0.0017	0.2956
Self-care ability	−0.0084	0.0024	0.0004
Region (reference group: northeast)			
Eastern	0.8437	0.0613	<0.0001
Central	0.5661	0.0634	<0.0001
Western	0.5792	0.0621	<0.0001
Gender (reference group: male)	0.0112	0.0326	0.7299
Educational attainment (reference group: schooled)	−0.0722	0.0339	0.0331
Marital status (reference group: married)	−0.1088	0.0368	0.0031
Primary source of income (reference group: self-generated income)	0.0718	0.0376	0.0564
Living arrangements (reference group: family)	0.1427	0.0396	0.0003
Quality of life	0.0369	0.0187	0.0485
Household financial status	−0.0122	0.0230	0.5963
Number of children born	−0.0196	0.0063	0.0019
Random effects variance			
Period	0.0973	0.0695	0.0809
Generation	0.0001	0.0008	0.4467
−2LogLikelihood	140273.8		

At the group level, the variance component associated with the period random effect is not statistically significant at the 5% level, indicating that period-specific random effects do not meaningfully contribute to variation in demand for expanded older population care services. Consequently, temporal heterogeneity at the period level appears to be statistically irrelevant once individual-level characteristics are controlled for.

#### 4.2.1 Age effect

Age does not exert a statistically significant linear effect on rural seniors’ demand for expanded older population care services. In the fixed-effects estimation of the HAPC model for expanded older population care service demand (Table 3), the coefficient on age is −0.0018 with a *p*-value of 0.2956, indicating no significant association between age and demand for expanded older population care services among rural seniors (24).

To further examine whether age-related differences in demand for expanded older population care services exhibit group heterogeneity, interaction terms between age and region, as well as between age and gender, are introduced. The estimation results are reported in Table 4. The findings show that neither regional heterogeneity nor gender heterogeneity produces statistically significant interaction effects. This indicates that rural older population individuals across different regions do not display stable age-related variation in demand for expanded older population care services. Similarly, no significant age-related differentiation is observed between male and female groups. Overall, after controlling for other covariates, neither regional context nor gender significantly moderates the relationship between age and demand for expanded older population care services.

**Table 4:** Group heterogeneity in age effects on demand for expanded older population care services among rural seniors.

Variable name	Regional heterogeneity			Gender heterogeneity		
	Coefficient	Standard deviation	*p*-value	Coefficient	Standard deviation	*p*-value
Age × region (eastern region)	−0.0045	0.0024	0.0624			
Age × region (central region)	0.0039	0.0028	0.1562			
Age × region (western region)	−0.0020	0.0025	0.4219			
Age × region (northeastern region)	−0.0082	0.0052	0.1117			
Age × gender (female)				−0.0014	0.0020	0.4840
Age × gender (male)				−0.0027	0.0024	0.2612
Control variables	Under control			Under control		

Taken together with the results for basic older population care services, these findings partially support H1. Specifically, the empirical evidence confirms that age is positively associated with demand for basic older population care services, but does not support the hypothesized negative relationship between age and demand for expanded older population care services.

#### 4.2.2 Period effect

**Figure 4:**
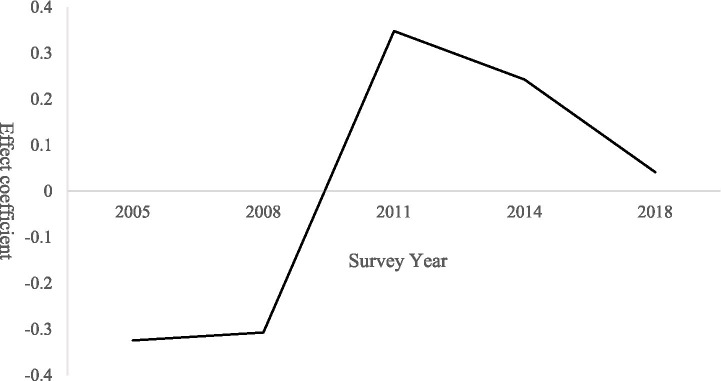
Period effects on demand for expanded older population care services among rural seniors.

The period-effect model indicates that demand for expanded older population care services follows a trajectory of initial growth followed by a subsequent decline. As shown in Figure 4, the period effect remains consistently negative between 2005 and 2008. During this stage, limited rural economic conditions constrained older population consumption capacity, with care needs concentrated primarily on basic subsistence security. Public service provision focused mainly on minimum livelihood, while expanded care services remained underdeveloped, resulting in the accumulation of latent demand.

From 2008 to 2011, demand increased sharply and reached a positive peak. This surge was driven by pro-farmer policies that enhanced rural seniors’ consumption capacity. The 2009 pilot of the New Rural Pension Scheme and the 2010 Older population Care Service System Development Plan explicitly incorporated expanded care services into their policy frameworks. At the same time, the growing prevalence of the “empty-nest” phenomenon amplified demand for social and recreational activities, leading to a rapid release of previously suppressed demand for expanded services (25).

Between 2011 and 2018, the period effect gradually declined but remained positive. This decline reflects the gradual satisfaction of both basic and expanded care needs following earlier investments in supporting infrastructure, such as convenience service outlets and cultural activity centers. In addition, increasing internet penetration in rural areas enabled older population individuals to access certain services online, reducing reliance on offline expanded care services and moderating demand intensity (25).

**Figure 5:**
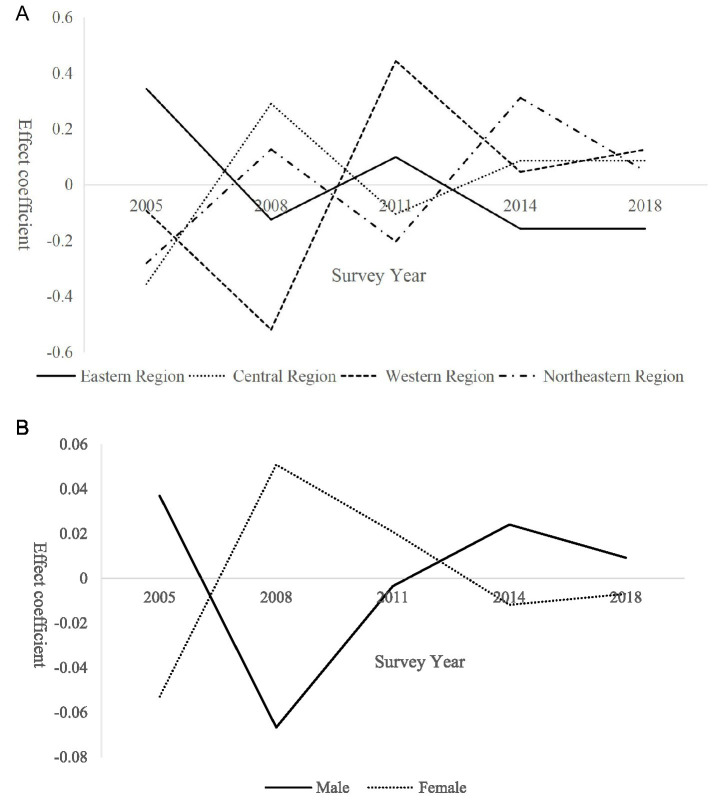
**(a)** Regional heterogeneity in period effects on demand for expanded older population care services among rural seniors. **(b)** Gender heterogeneity in period effects on demand for expanded older population care services among rural seniors.

The results of group heterogeneity tests for period effects on demand for expanded older population care services are presented in Figure 5. Figure 5a illustrates regional heterogeneity in period effects. In the eastern region, the effect is relatively high in 2005 but declines rapidly thereafter. This pattern reflects the region’s early economic development, which facilitated early emergence of demand for expanded services, followed by demand attenuation due to accelerated urbanization and a higher incidence of older population migration. In the central region, the effect rises sharply between 2005 and 2008 and then fluctuates downward. As a major labor-exporting region, the rapid emergence of “empty-nest” households initially released strong demand for social and recreational services, which was later partially satisfied by improvements in county-level commercial and cultural facilities.

In the western region, the effect drops to a low point between 2005 and 2008 before rebounding markedly. This rebound reflects the release of long-suppressed demand resulting from earlier economic constraints and limited service supply, which was subsequently alleviated by poverty alleviation initiatives and infrastructure investments. In contrast, the northeastern region exhibits a sustained upward trend in demand since 2005. Persistent rural depopulation weakened family-based caregiving, while slow expansion of expanded care services amid economic restructuring led to the continuous accumulation of demand pressure (25).

Figure 5b presents gender heterogeneity in period effects. Among men, the effect follows a pattern of sharp decline, rebound, and gradual decline. Between 2005 and 2008, the effect falls rapidly, reflecting the tendency for men’s recreational and social needs to be overlooked when they were primarily engaged as household breadwinners. From 2008 to 2011, improvements in rural economic conditions and expanded older population care policies released latent leisure-related demand. Subsequently, aging and declining health reduced participation in expanded care activities, leading to a gradual decline in the effect.

Among women, the period effect shows a pattern of continuous decline. Although women’s social and recreational needs might be expected to increase with the persistence of “empty-nest” households, long-standing caregiving responsibilities, stronger reliance on family-based social interactions, and limited supply of public recreational services in rural areas constrained the effective realization of demand for expanded services.

Taken together, analysis of period effects for both basic and expanded older population care services indicates that overall demand for socialized older population care services among rural seniors increased over the 2005–2018 period. This finding supports the core assertion of H2 that demand for older population care services exhibits a positive temporal evolution. However, both types of services display pronounced fluctuations over time, reflecting the combined influence of policy interventions, economic conditions, and structural transformations in rural society.

#### 4.2.3 Cohort effect

**Figure 6:**
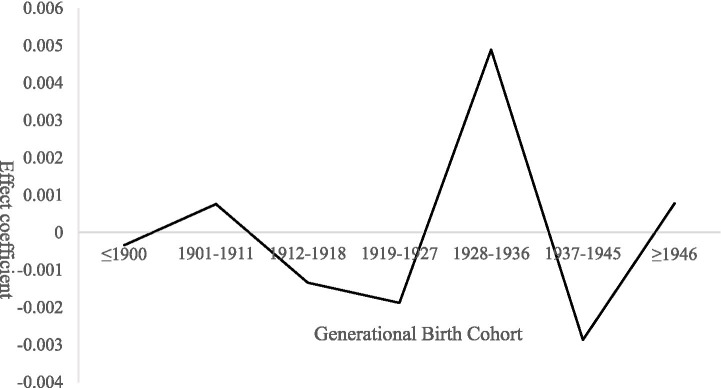
Cohort effects on demand for expanded older population care services among rural seniors.

The cohort-effect model indicates that demand for expanded older population care services follows an overall W-shaped fluctuation pattern across birth cohorts, as illustrated in Figure 6. The estimated cohort coefficients display marked variation by generational group (26). For cohorts born ≤1911, the effect is slightly positive. This generation spent most of their lives in a traditional agrarian society characterized by relatively intact family structures and high levels of household self-sufficiency. Daily shopping was largely satisfied through local markets, and social interaction centered on kinship networks, rendering demand for expanded services largely latent.

For the 1912–1927 cohort, the cohort effect declines steadily into negative territory. This pattern reflects prolonged exposure to wartime instability and the early transition toward collective economic systems, which resulted in material shortages and underdeveloped public services in rural areas, thereby suppressing demand for expanded older population care services over an extended period. In contrast, the 1928–1936 cohort exhibits a pronounced surge, reaching the highest positive effect. This cohort entered old age during the post-2010 period, when rural older population care systems were substantially improved and pro-farmer policies enhanced consumption capacity, leading to the concentrated release of previously suppressed demand for shopping and recreational services. The cohort effect for individuals born between 1937 and 1945 declines markedly. Having grown up during periods of social turmoil, this generation generally holds conservative attitudes toward aging and demonstrates lower acceptance of newly emerging expanded care services. For cohorts born ≥1946, the effect shows a modest rebound. Although this group benefited from expanded service provision, a substantial proportion relocated to urban areas to live with their children, weakening demand for locally provided expanded services in rural areas.

**Figure 7:**
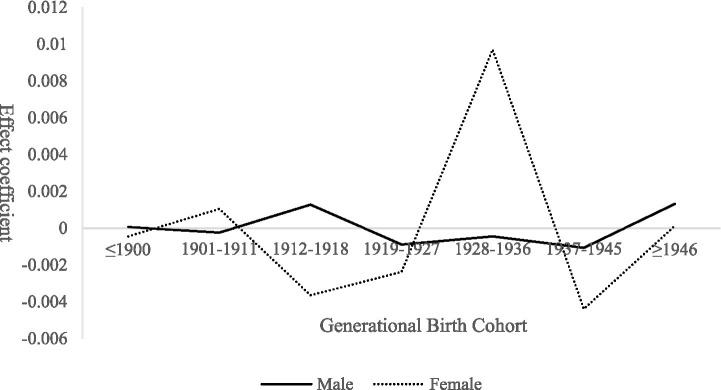
Gender heterogeneity in cohort effects on demand for expanded older population care services among rural seniors.

The results of group heterogeneity analysis of cohort effects are presented in Figure 7. Among males, the cohort effect exhibits relatively gentle fluctuations. For those born ≤1900, the effect is close to zero, followed by minor oscillations across cohorts, dipping to approximately zero for the 1937–1945 cohort before slightly rebounding thereafter. This pattern corresponds to gendered family roles: earlier male cohorts, as primary household breadwinners, often had their recreational and consumption-related needs overlooked; middle cohorts experienced fluctuations driven by broader social and economic transformations; and later cohorts saw modest demand release as service availability expanded, although the effect remained limited due to migration to urban areas.

In contrast, cohort effects among females display substantially greater volatility. The effect is slightly positive for those born ≤1900, drops sharply for the 1912–1918 cohort, and then rises to a pronounced peak for the 1928–1936 cohort before declining rapidly thereafter. For earlier cohorts, women’s primary caregiving responsibilities within the family suppressed the expression of their own expanded care needs. During mid-period cohorts, wartime disruption and resource scarcity further constrained participation in recreational activities. For later cohorts, older population years coincided with improved rural service provision, and living in “empty-nest” households intensified demand for social and cultural engagement, producing the observed peak. However, with advancing age and declining health, participation in expanded services decreased sharply.

Taken together, analysis of cohort effects for both basic and expanded older population care services reveals clear differentiation. While no statistically significant cohort differences are observed in demand for basic care services among rural older population across birth cohorts, demand for expanded care services exhibits pronounced cohort-specific fluctuations. These variations are closely linked to early-life social environments and population policy contexts. Accordingly, the findings partially support H3, confirming that generational factors influence older population care service demand. However, the assumption that generational factors exert uniform effects on both basic and expanded care service demand is not supported by the empirical evidence.

## 5 Research findings and discussion

### 5.1 Research findings

This study employs a hierarchical age–period–cohort (HAPC) model and data from the 2005–2018 China Longitudinal Healthy Longevity Survey (CLHLS) to systematically examine the evolutionary patterns and underlying mechanisms of demand for socialized older population care services among rural seniors. Several key findings emerge.

First, with respect to age effects, age exerts a significant and positive influence on demand for basic older population care services. As rural seniors grow older and their self-care capacity declines, demand for fundamental services—such as daily living assistance and medical care—continues to increase. This age effect exhibits pronounced heterogeneity across gender and region (particularly in eastern, central, and western China). In contrast, age does not exert a significant linear effect on demand for expanded older population care services.

Second, regarding period effects, demand for both basic and expanded older population care services follows a fluctuating upward trajectory characterized by an initial increase followed by a decline. The period from 2008 to 2011 represents a critical inflection point, during which demand was rapidly released as a result of concentrated policy implementation. Regional heterogeneity in period effects is substantial, while gender heterogeneity is more evident for expanded care services.

Third, concerning cohort effects, demand for basic older population care services shows no significant generational differentiation overall, although gender-specific fluctuations are observed within cohorts. In contrast, demand for expanded older population care services displays a distinct “W-shaped” cohort pattern, peaking among individuals born between 1928 and 1936. Later-born cohorts demonstrate greater acceptance of expanded services, reflecting the influence of improved early-life social environments and evolving population policies. In addition, several control variables—particularly self-care ability and region—exert significant effects on service demand, underscoring its multidimensional and differentiated nature.

Methodologically, this study advances existing research by moving beyond traditional static analyses and constructing a three-dimensional dynamic framework that integrates age, period, and cohort effects. By identifying both the independent influences and interactive mechanisms among these temporal dimensions, the study validates the applicability of life course theory in the context of rural older population care demand. It further provides a rigorous quantitative approach for disentangling the interplay between “policy-driven” institutional forces and “individual choice” dynamics.

From a policy perspective, the findings offer targeted guidance for optimizing rural older population care service systems. They highlight the need to tailor service provision to heterogeneous age and cohort groups, formulate region-specific policy interventions based on local development conditions, and recognize the pivotal role of policy in activating and shaping care demand. Overall, this research delineates practical pathways for refining older population care policies, addressing supply–demand mismatches, and improving service accessibility, thereby contributing to more effective responses to the challenges of population aging in rural China.

### 5.2 Discussion

This study employs a hierarchical age–period–cohort (HAPC) model to effectively circumvent the multicollinearity problem inherent in traditional APC frameworks. Through hierarchical specification, the model separately identifies age, period, and cohort effects, enabling precise quantification of the independent influences of individual life-course processes, macro-level policy dynamics, and generational characteristics on older population care service demand. In doing so, the study provides a rigorous analytical tool for dynamically examining the evolution of demand. The use of China Longitudinal Healthy Longevity Survey (CLHLS) panel data spanning 2005–2018, comprising 30,585 observations across 23 provinces and municipalities, ensures strong representativeness and enhances the robustness of the empirical findings.

Nevertheless, several methodological limitations remain. First, the HAPC framework does not fully capture potential nonlinear interactions among explanatory variables, which may lead to underestimation of complex behavioral responses. Second, the model does not explicitly incorporate macro-level contextual indicators—such as regional economic development, fiscal capacity, or service infrastructure—which may affect the precision of certain estimated effects. These constraints suggest scope for methodological extension in future research.

Empirically, the study confirms the positive influence of age on demand for basic older population care services and highlights the important role of period effects, consistent with the dual forces of accelerating rural population aging and sustained policy expansion. However, an unexpected finding emerges: age does not exert a significant effect on demand for expanded older population care services. This divergence from theoretical expectations may reflect the overall insufficiency of expanded service supply in rural areas, which constrains the effective expression of latent demand across age groups. Moreover, cohort effects are significant only for expanded care services, indicating that basic care constitutes a rigid and universally necessary demand across generations, whereas expanded care needs are more sensitive to historical context, early-life environments, and policy evolution. The presence of pronounced regional and gender heterogeneity further demonstrates that rural older population care demand is inherently differentiated, underscoring the limitations of a uniform, one-size-fits-all service provision strategy.

Several data-related limitations also warrant attention. As a publicly available dataset, the CLHLS prioritizes health status and survival outcomes, resulting in relatively coarse measurement of older population care service demand. The absence of certain key variables further constrains the comprehensive identification of influencing factors. In addition, the data end in 2018 and therefore do not capture recent policy initiatives and structural changes—such as rural revitalization strategies and digital village development—implemented after 2020, limiting the study’s ability to reflect current demand patterns. Finally, the analysis focuses exclusively on the demand side, without incorporating supply-side information, preventing a complete assessment of supply–demand matching in rural older population care services.

Future research will address these limitations by incorporating more recent survey data, enriching macro-level contextual variables, and integrating supply-side indicators. By jointly analyzing demand and supply dimensions, subsequent studies can better identify emerging care needs among rural seniors and enhance the policy relevance and practical applicability of research conclusions.

## Data Availability

The datasets presented in this study can be found in online repositories. The names of the repository/repositories and accession number(s) can be found at: https://opendata.pku.edu.cn/dataverse/CHADS.

## Footnotes

1 National Bureau of Statisticshttps: //www.stats.gov.cn/zs/tjws/tjbz/202301/t20230101_1903949.html
